# Fructose-Bisphosphate Aldolase A Is a Potential Metastasis-Associated Marker of Lung Squamous Cell Carcinoma and Promotes Lung Cell Tumorigenesis and Migration

**DOI:** 10.1371/journal.pone.0085804

**Published:** 2014-01-23

**Authors:** Sha Du, Zhuzhu Guan, Lihong Hao, Yang Song, Lan Wang, Linlin Gong, Lu Liu, Xiaoyu Qi, Zhaoyuan Hou, Shujuan Shao

**Affiliations:** 1 Department of Histology and Embryology, Dalian Medical University, Liaoning, China; 2 Institute of Cancer Stem Cell, Dalian Medical University Cancer Center, Liaoning, China; 3 Liaoning Key Laboratory of Proteomics, Dalian Medical University, Liaoning, China; University of South Alabama Mitchell Cancer Institute, United States of America

## Abstract

Fructose-bisphosphate aldolase A (ALDOA) is a key enzyme in glycolysis and is responsible for catalyzing the reversible conversion of fructose-1,6-bisphosphate to glyceraldehydes-3-phosphate and dihydroxyacetone phosphate. ALDOA contributes to various cellular functions such as muscle maintenance, regulation of cell shape and mobility, striated muscle contraction, actin filament organization and ATP biosynthetic process. Here, we reported that ALDOA is a highly expressed in lung squamous cell carcinoma (LSCC) and its expression level is correlated with LSCC metastasis, grades, differentiation status and poor prognosis. Depletion of ALDOA expression in the lung squamous carcinoma NCI-H520 cells reduces the capabilities of cell motility and tumorigenesis. These data suggest that ALDOA could be a potential marker for LSCC metastasis and a therapeutic target for drug development.

## Introduction

Squamous cell carcinoma (SCC) is the second most common type of lung cancer accounting for about 30% of all lung cancers [1]. When diagnosed early, lung SCC (LSCC) is well curable by surgical excision. However, most of LSCC patients encounter high rate of recurrence for metastasis and resistance to existing chemotherapeutic agents after resection. Therefore, in order to reduce mortality of LSCC, it is necessary to identify molecular markers for early diagnosis and elucidate the biochemical mechanism governing the processes of recurrence and metastasis as well as therapeutic resistance.

A proteomic approach using fluorescent dye-labeled proteins coupled with two-dimensional gel electrophoresis (2-DIGE) and mass spectrometric (MS) analysis has been widely applied to identify differentially expressed proteins between normal and tumor specimens [2]. These differentially expressed proteins could either serve as molecular markers for diagnosis or lead to understanding the molecular mechanisms of metastasis and therapeutic resistance. By employing the 2-DIGE and MS approaches, we compared the protein profiles between clinical metastatic, non-metastastic LSCC tissues and adjacent normal lung tissues, and identified a number of differentially expressed proteins participating in many biological functions such as cell signaling regulation, carbohydrate metabolism, molecular chaperones, and protein synthesis. Among these protein candidates, we were particularly interested in fructose-bisphosphate aldolase A (ALDOA), an key enzyme in glycolysis responsible for catalyzing the reversible conversion of fructose-1,6-bisphosphate to glyceraldehydes-3-phosphate and dihydroxyacetone phosphate [3].

ALDOA is one of the three aldolase isozymes (A, B, and C), encoded by three different genes. These aldolases are differentially expressed during development. ALDOA is highly expressed in the developing embryo and in adult muscle [3]. ALDOA contributes to various cellular functions and biological process related to muscle maintenance, regulation of cell shape and mobility, striated muscle contraction, actin filament organization and ATP biosynthetic process [4]–[13]. ALDOA deficiency is associated with myopathy and hemolytic anemia [14]–[16]. Notably, ALDOA has been found highly expressed in a variety of malignant cancers, including human lung squamous [17]–[18], renal cell [19] and hepatocellular carcinomas [20]. However, none of these reports examined the involvement of ALDOA in LSCC progression and metastasis.

In this study, we reported that ALDOA is highly expressed in LSCC and its expression level is correlated with LSCC metastasis. Further, we demonstrated that depletion of ALDOA in lung cancer cells reduces its tumorigenicity and capability of migration. These observations suggest that ALDOA is a potential biomarker of LSCC metastasis and play important role in LSCC progression and metastasis.

## Materials and Methods

### Samples Preparation and Proteomic Analysis

Seven pairs of matched primary LSCC samples (6 male and 1 female aging from 36 to 67 years old with an average age of 55 years old) were obtained from the Department of Thoracic Surgery of the First Affiliated Hospital of Dalian Medical University, China. Three pairs are non-metastatic and 4 pairs are metastatic. No patients received preoperative radiotherapy and chemotherapy. The study was approved by the Ethic and Research Committees of Dalian Medical University and was conducted in accordance with the Declaration of Helsinki Principles. The patients thoroughly understood the collecting process and purpose of using the specimens, and signed “informed consents-specimen collection. The fresh samples from tumor and normal tissues (>5 cm away from the lesion) were snap-frozen and stored at −80°C. The pathological diagnosis was done to confirm that tumor specimens were real SCC tissues. Surgery follow-ups were conducted to each patient at an interval of 12 months for 3 years.

To prepare protein extracts, the tissues were homogenized in buffer containing 7 M urea, 2 M thiourea, 4% CHAPS, 30 mM Tris, and a cocktail of protease inhibitors (GE Healthcare) and the supernatants were collected by centrifugation at 12,000 g for 15 min at 4°C. 50 ug of pooled protein extracts was labeled with Cy2 as the internal standard control, Cy3 and Cy5 were used to label experimental samples. The resulting samples were resolved bi-dimensionally on 12.5% SDS-PAGE gels. Images were acquired using the fluorescence scanner (GE Healthcare) at excitation wavelengths of 488/520 nm, 532/580 nm or 633/670 nm, respectively. The image analysis was processed using DeCyder 6.5 (GE Healthcare). BVA software module was used for matching spots between gels and average abundance and statistics calculation. The protein abundance was represented by the volume ratio of samples versus standards and the proteins of interest with an average ratio more than 1.50 or less than −1.50 were selected for mass spectrometry analysis.

### Western Blot, immunofluorecence and LSCC tissue microarrays

Protein extracts (50 µg) were resolved on 12% SDS-PAGE gels, transferred to nitrocellulose membranes (0.45 µm), and immunoblotted with rabbit anti-human ALDOA antibody (HPA004177, Sigma; 1∶1500) or mouse anti-human β-actin monoclonal antibody (1∶4000). Images were developed with ECL (GE HealthCare, USA). Immunofluorecence staining of ALDOA protein was performed in NCI-H520 cells, and images were taken using confocal microscopy.

The LSCC tissue microarrays contain 75 matched pairs of specimens (Chaoxin Biotechnology Co., Shanghai, China). Strict pathological diagnoses and post-operative follow-ups were performed for all patients. Thirty-two cases are from metastatic LSCC patients and 43 cases are from non-metastatic LSCC patients. IHC staining was performed to detect the expression of ALDOA.

Sections were scored as positive if epithelial cells or tumor cells showed a staining reaction in the cytoplasm and/or the nucleus. A quantitative score was given by estimating the percentages of positive cells: 0, (0 to 5%); 1, (5% to 25%); 2, (25% to 50%); 3, (50% to 75%); and 4, (75% to 100%). The intensity of positive staining was given scores as negative (0), pallide-flavens grains (1), buffy grains (2) and brown-black grains (3) respectively. Finally, total scores (0–12) for each samples were determined by combination of the quantitative scores time the intensity scores. The score 0 to 4, 5 to 8, and 9 to 12 indicated ALDOA expression as negative, positive and strongly positive.

### Cell Culture and Stable Knockdown of ALDOA in NCI-H520

The human lung squamous cell carcinoma NCI-H520 cell line was maintained in RPMI 1640 medium (Invitrogen, USA) supplemented with 10% fetal bovine serum (FBS).

The pGPU6/GFP/Neo vector containing shRNAs specific to human ALDOA were transfected into NCI-H520 cells using Lipofectamine 2000 (Invitrogen) and the resulting cells were selected with G418 (400 µg/ml) to establish clones of stable depletion of ALDOA.

### Cell Migration assays, soft agar colony formation assays and xenografting nude mice

Cells were seeded in 10-cm petri dishes at a density of 1×10**^6^** cells/ml and grown to approximately 90% confluency. The cells were cut with a sterile 200-µl pipette tip and the resulting cells were continually cultured in serum-free medium and were photographed at 0, 8 and 24 hs post-scratching.

Cell migration assays were performed in the transwell plates with an 8-µm pore size membrane (Corning, New York, NY). 5×10**^5^** cells were put into the upper chamber and medium containing 10% FBS as attractant was placed in the lower chamber. After 24 hours of incubation the cells on the upper surface of the filter were wiped out with cotton swabs and the migrated cells were fixed in 70% methanol and stained with 0.1% crystal violet. The assays were performed in triplicates and at least 6 unbiased fields were counted per filter. The mean cell numbers and standard deviations were calculated.

Six weeks old male nude mice (Inspection No: 0002858) were purchased and allowed to acclimate one week in the animal facility. NCI-H520 cells and derivatives (10^7^ cells in 200 µL medium) were prepared and injected subcutaneously into the left flank of the mice (three mice per group). The mice were checked daily for tumor growth, and sacrificed 8 weeks after injection. The isografted tumors were thoroughly examined.

### Statistical analysis

All statistical analyses were performed using SPSS 12.0 software. Data were presented as mean ± standard deviation (S.D.). The chi-square test and Fisher's exact test were used to analyze the association between ALDOA expression and clinicopathologic features of SCC. Kaplan-Meier methods with the log-rank test were used to estimate differences in survival curves among LSCC patients. Comparisons among all groups were performed with the one-way analysis of variance (ANOVA) test and Student Newman Keuls method. P-values less than 0.05 were considered as statistically significant.

## Results

### ALDOA is highly expressed in LSCC metastasis

**Figure 1:**
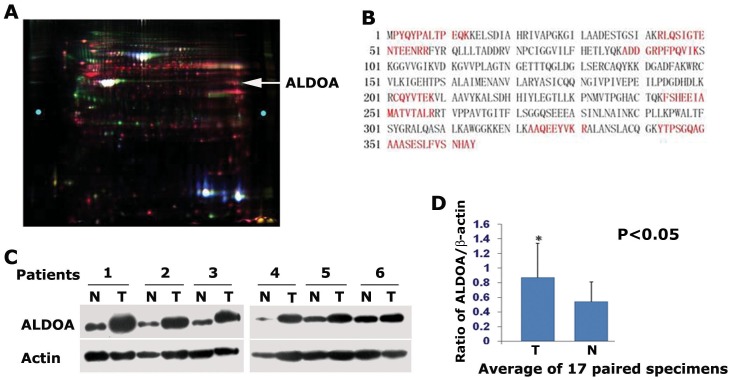
ALDOA is highly expressed in lung squamous carcinoma. (A) Proteomic analysis of the differentially expressed proteins from normal tissue and LSCC metastasis. Strictly selected 7 pairs of the matched LSCC specimens were subjected for 2D-DIGE and MS proteomic analysis. ALDOA was up-regulated 3.12-fold in metastatic LSCC tissues and 1.77-fold in non-metastatic LSCC tissues compared to adjacent normal tissues (the pointing arrow shows the ALDOA spots). (B) The amino acid residues highlighted in red were those detected by MS/MS analysis and counted for 36% sequence coverage of ALDOA. (C) Western blotting analysis of the ALDOA protein expression in 17 pairs of LSCC and matched adjacent normal tissues. Higher expression of ALDOA was observed in most of LSCC tissues examined. Data from 6 pairs of specimens were shown here. (D) Average of the ALDOA protein from 17 pairs of matched specimens. The relative expression values of ALDOA were 0.87±0.47 in carcinoma tissues and 0.54±0.27 in normal tissues. The level of Actin was used as control for normalization.

To identify differentially expressed proteins from LSCC, strictly selected 7 pairs of the LSCC specimens from well documented patients were subjected to 2D-DIGE and MS proteomic analysis. Of the protein identified, 63 proteins were found more than 1.5- or less than −1.5-fold difference in metastatic or non-metastatic SCC tissues compared to the adjacent normal tissues. ALDOA was up-regulated 3.12-fold in metastatic LSCC tissues and 1.77-fold in non-metastatic LSCC tissues (Figure 1A). The amino acid residues highlighted in red were those detected by MS analysis and counted for 36% sequence coverage of ALDOA (Figure 1B).

We next carried out western blotting assays to examine the ALDOA protein expression in 17 pairs of LSCC and matched adjacent normal tissues. Higher expression of ALDOA was observed in most of LSCC tissues examined (Figure 1C). The level of Actin was used as a control for normalization. The relative expression values of ALDOA were 0.87±0.47 in carcinoma tissues and 0.54±0.27 in normal tissues, respectively (Fig. 1D).

### ALDOA is predominately localized in cytoplasm

**Figure 2:**
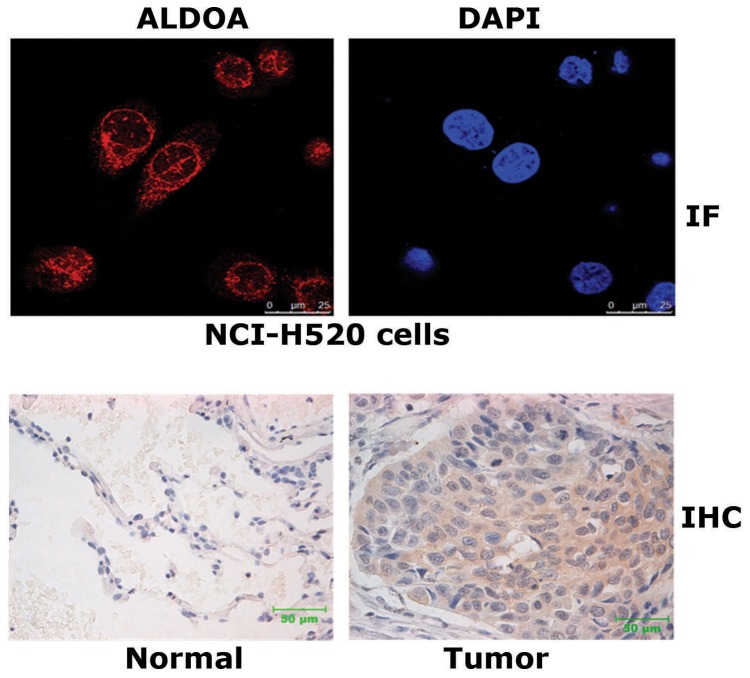
ALDOA is a major cytoplasmic protein. (**A**) Endogenous ALDOA was detected by immunofluorescence staining using antibody specific to ALDOA in NCI-H520 cells. Cellular ALDOA protein (red) and nuclear condensation (blue, DAPI) were examined by using a Leica confocal SP5 microscopey. (B) IHC assays of ALDOA were performed in paired LSCC tissues vs adjacent normal tissues.

To examine the subcellular localization of the endogenous ALDOA, we first performed immunofluorecence staining assays in NCI-H520 cells using antibody specific to ALDOA. Images taken by confocal microscopy showed that ALDOA was predominately distributed in cytoplasm, and in the nucleus ALDOA formed sporadic bright dots (Figure 2A). Next, we performed IHC assays to examine the localization of ALDOA in LSCC tissues. Consistently, strong staining of ALDOA was observed in the cytoplasm of the tumor cells (Figure 2B).

### High expression of ALDOA is correlated with LSCC metastasis, tumor grades and differentiation status

**Figure 3:**
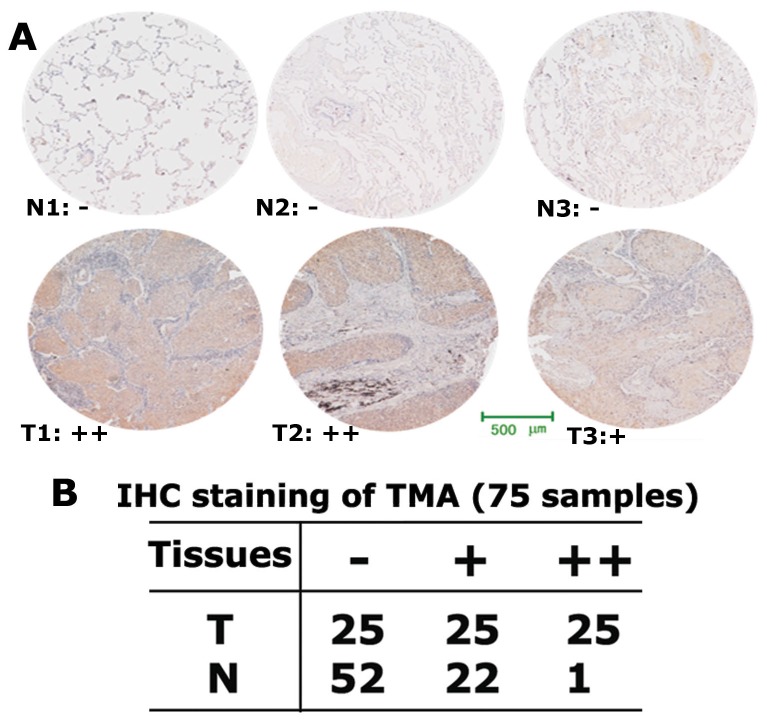
High expression of ALDOA is highly expressed in LSCC metastasis. (**A**) Tissue microarray (TMA) analysis of ALDOA expression in 75 pairs of matched LSCCs and adjacent normal tissues. IHC staining assays were performed to examine ALDOA protein expression and three pairs were shown as representatives. (B) In normal tissues 22 cases (29.3%) were measured as positive (+), and 1 case (1.3%) was strong positive (++). In LSCC tissues 25 cases (33.3%) were measured as positive (+) and 25 cases (33.3%) were strong positive (++).

To analyze the clinical relevance between high expression of ALDOA and LSCC, we performed immunochemistry staining to examine ALDOA protein expression in a LSCC tissue microarray (TMA) containing 75 pairs of matched LSCCs and adjacent normal tissues. Among which 32 pairs are from metastatic LSCC patients and 43 pairs are from non-metastatic LSCC patients. The intensity of the positive stain of TMA was measured and quantified as positive (+) or strong positive (++) (Figure 3A). In normal tissues 22 cases (29.3%) were measured as positive (+), and 1 case (1.3%) was strong positive (++). Strikingly, in LSCC tissues 25 cases (33.3%) were measured as positive (+) and 25 cases (33.3%) were strong positive (++) (Figure 3B).

We further analyzed if there are any correlations between the protein level of ALDOA in LSSC tissues and tumor metastasis, grade and differentiation status. ALDOA shows a higher positive rate (84.4%, 27 out of 32 cases) of expression in metastatic LSSC tissues than that of non-metastatic LSSC tissues (53.5%, 23 out of 43 cases). Moreover, 19 cases showed strong positive ALDOA stain in metastatic LSCC tissues, while only 6 cases showed strong positive stain in non-metastatic LSCC tissues (Table 1). Similarly, high ALDOA expression was observed with increasing tumor grades and differentiation status.

**Table 1:** Summary of the TMA analysis of ALDOA expression.

ALDOA level	Metastasis		Tumor grades				Differentiation		
	no	yes	I	II	III	IV	low	middle	high
**−**	20	5	15	6	4	0	7	13	3
**+**	17	8	9	5	6	0	2	22	3
**++**	6	19	3	17	8	2	4	17	4
**Positive (%)**	53.5	84.4	44.4	78.6	77.8	100	46.2	75	70
**χ2**	7.876		9.786				4.072		
**p value**	0.005		0.02				0.13		

Correlations of the protein level of ALDOA in LSSC tissues and tumor metastasis, grade and differentiation status. Of the total 75 specimens on TMA examined, 32 pairs are from metastatic LSCC patients and 43 pairs are from non-metastatic LSCC patients. The intensity of the positive stain of TMA was measured and quantified as positive (+) or strong positive (++).

**Figure 4:**
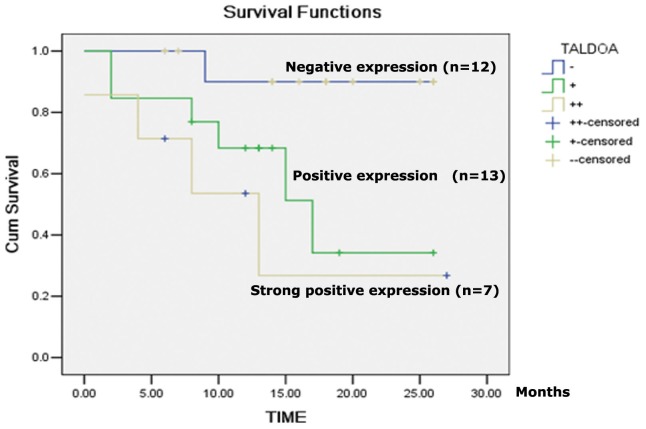
Expression level of ALDOA protein is negatively correlated with survival rates and prognosis of LSCC patients. Thirty-two out of the 75 cases on TMA met the requirement for the Kaplan-Meier method analysis. −: negative expression; +: positive expression; ++: strong positive expression.

We next investigated the correlation of ALDOA expression with patients' prognosis. Thirty-two out of the 75 cases on TMA met the requirement for the Kaplan-Meier method analysis. The results indicated that patients having high expression of ALDOA displayed low survival rate (Figure 4). Taken together, high expression of ALDOA is associated with LSCC metastasis and poor prognosis.

### ALDOA is required for migration of the lung cancer cell NCI-H520

**Figure 5:**
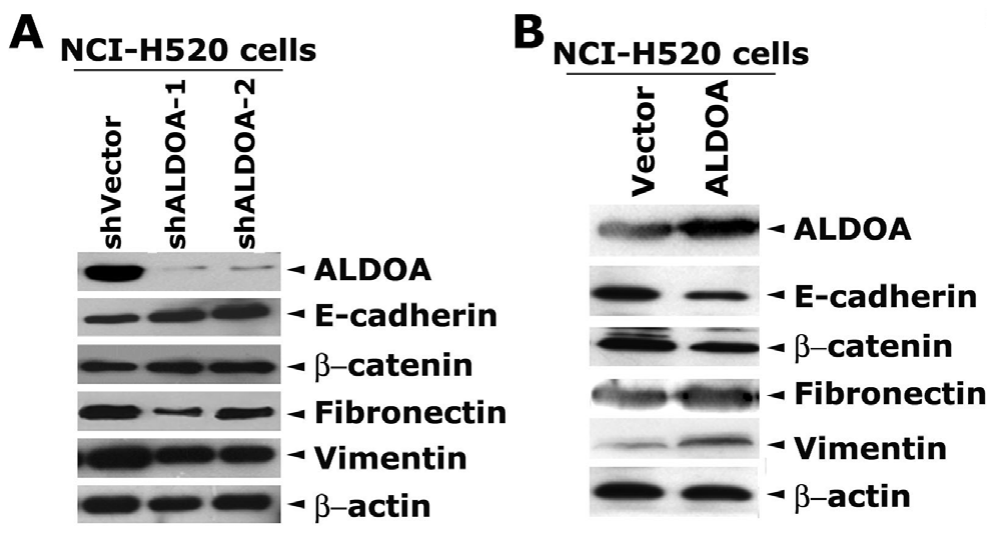
ALDOA affects the expression of epithelial and mesenchymal markers in NCI-H520 cells. (A) Depletion of ALDOA in NCI-H520 cells results in reversal expression of epithelial and mesenchymal markers. Two independent shRNAs specific to ALDOA were stably expressed in NCI-H520 cells. Western blot assays showed that the two shRNAs efficiently decreased ALDOA protein level, and concomitantly the epithelial markers E-cadherin and β-catenin were increased while the mesenchymal markers Fibronectin and Vimentin were decreased. (B) Overexpression of ALDOA decreased E-cadherin and β-catenin and concomitantly increased Fibronectin and Vimentin. ALDOA protein was expressed in NCI-H520 cells via transcient transfection of palsmids encoding human ALDOA. The parental vector was used as a control. The expression of various proteins was analyzed by western blot assays.

To examine if ALDOA is required for lung cancer cell migration, we depleted ALDOA in NCI-H520 cells by stably expressing two independent shRNAs specific to ALDOA. Western blot assays showed that the two shRNAs efficiently decreased ALDOA protein level (Figure 5A), and concomitantly depletion of ALDOA resulted in an up-regulation of epithelial markers E-cadherin and β-catenin, and a down regulation of mesenchymal markers Fibronectin and Vimentin. Conversely, overexpression of ALDOA in NCI-H520 cells decreased E-cadherin and β-catenin, and concomitantly increased Fibronectin and Vimentin (Figure 5B). These observations suggest that ALDOA may induce epithelial-mesenchymal transition and promote cell migration.

**Figure 6:**
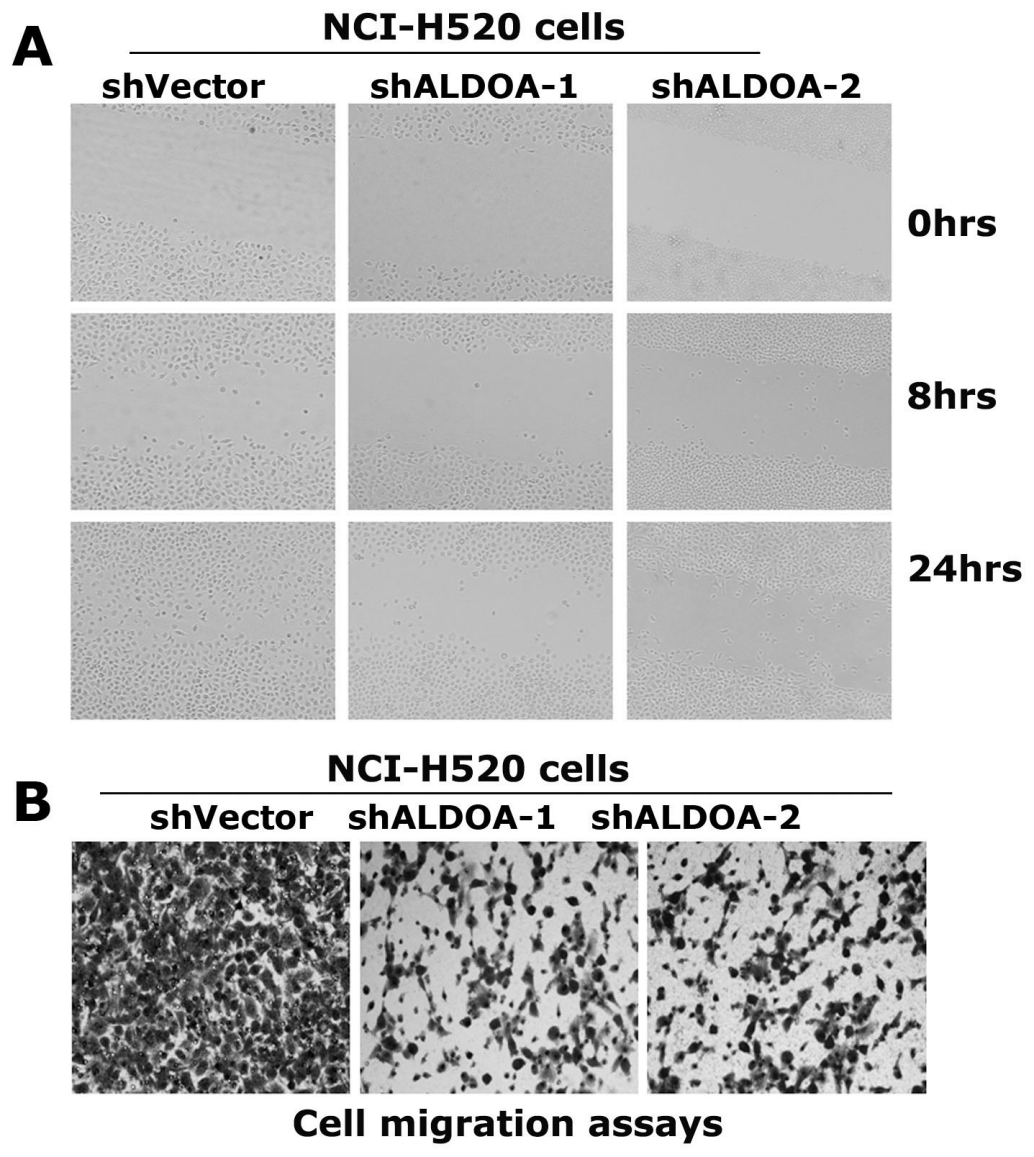
ALDOA is required for migration of the lung cancer cell NCI-H520. (A) Wound-healing assays of the resulting NCI-H520 cells in cultured plates. Cell were grown in petri dishes to 90% confluency and cut with a sterile 200-µl pipette tip. The resulting cells were continually cultured in serum-free medium and were photographed at 0, 8 and 24 hs post-scratching. (B) Cell migration assays using the transwell assays kits. 5×10**^5^** cells per well were plated in the upper chambers of transwell plates with an 8-µm pore size membrane and media containing 10% FBS was placed in the lower chamber. After 24 hours of culture the cells on the upper surface of the filter were removed. The migrated cells were fixed in 70% methanol and stained with 0.1% crystal violet. The assays were performed in triplicates and at least 6 unbiased fields were counted per filter.

We next carried out wound-healing assays in these established NCI-H520 cell derivatives stably expressing vector or shRNAs specific to ALODA. At 8 hrs after scratching, NCI-H520-shVector cells apparently migrated to the wound area, and at 24 hrs the wound area was well covered by the cells. In contrast, no apparent cell migration was observed in NCI-H520-shALDOA cells at 8 hrs, and the wound area was only partially covered by cells at 24 hrs (Figure 6A).

To further examine the effect of depletion of ALDOA on cell motility, the migration potential of the resulting cells was assayed using transwell plates. Migrated cells were monitored using an inverted microscopy. Depletion of ALDOA apparently reduced NCI-H520 cells migration (Figure 6B). Taken together, these data indicate high expression of ALDOA is required for NCI-H520 cells to migrate effectively.

### Depletion of ALDOA reduces the tumorigenicity of NCI-H520 cells

**Figure 7:**
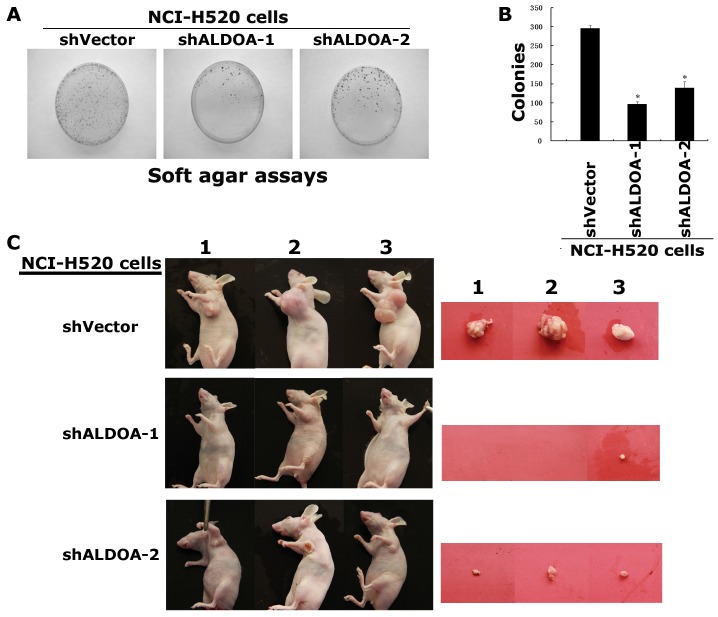
Depletion of ALDOA reduces the tumorigenicity of NCI-H520 cells. (A) Soft agar assays. The NCI-H520-siALDOA cells and NCI-H520-vector cells were suspended in single cell and cultured in soft agar to evaluate anchorage-independent colony formation. The experiments were done in triplates and repeated twice. (B) Data shows the average of the colonies formed by these NCI-H520 cells in triplicates. (C) The NCI-H520-shALDOA cells did not grow or only grow into very small tumors in the nude mice.

To examine the effect of ALDOA on the tumorigenicity, suspensions of single cancer cells of the NCI-H520-shALDOA cells and NCI-H520-shVector cells were cultured in soft agar to evaluate anchorage-independent colony formation. The colonies formed by these NCI-H520-shALDOA cells were significantly decreased in colony number, compared with NCI-H520-shVector cells (Figure 7A, B). Consistently, when transplanted subcutaneously into the nude mice, the NCI-H520-siALDOA cells did not grow or only grow into very small tumors compared with that of NCI-H520-shVector cells (Figure 7C, D).

## Discussion

In the present study, we identified the glycolytic enzyme ALDOA was highly expressed in metastatic LSCC, and its express is highly correlated with LSCC metastasis, tumor grade and differentiation status. We further demonstrated that depletion of ALDOA expression in NCI-H520 cells reduced the capabilities of cell motility and tumorigenesis. These data suggest that ALDOA could be a potential marker for LSCC metastasis and a potential therapeutic target for drug development.

A typical feature of tumor cells is highly active glycolysis associated to an inhibition of apoptosis. As first stated by Warburg, cancer cells need to activate glycolysis to proliferate despite the presence of oxygen because glycolysis provides most of the building blocks required for massive cell proliferation [21]. ALDOA is a ubiquitous glycolytic enzyme that drives the glycolytic metabolic pathway in mammalian cells and is predominantly expressed in adult muscle tissue. Overexpression of ALDOA is observed in various cancers including lung, renal cell and hepatocellular carcinoma, suggesting enhanced glycolysis in these cancer cells [17]–[20].

We also observed that depletion of ALDOA results in an up-regulation of epithelial markers and a down regulation of mesenchymal markers, suggesting ALDOA is required for maintaining the mesenchymal morphology, a characteristic of migrating cells. Accordingly, our results indicate that overexpression of ALDOA was significantly relevant to high degree of metastasis and low degree of pathologic staging, as well as low survival rate and poor prognosis. These findings suggest ALDOA could be a potential marker for LSCC metastasis, prognosis prediction and as a target for clinical treatment of LSCC.
