# Expression of Concern: Fructose-Bisphosphate Aldolase A Is a Potential Metastasis-Associated Marker of Lung Squamous Cell Carcinoma and Promotes Lung Cell Tumorigenesis and Migration

**DOI:** 10.1371/journal.pone.0285076

**Published:** 2023-04-24

**Authors:** The PLOS ONE Editors

Following the publication of this article [1], the corresponding authors contacted the journal to request correction of errors in multiple figures. Additional issues were also identified during assessment. Specifically,

- The 2D gel electrophoresis image in Figure 1A in [1] appears similar to Figure 1A in [2], Figure 2A in [3], and Figure 1A in [4], and the patient cohort described in each article appears to be similar.
- The shVector panels in Figure 6A of [1] are similar to the NCI-H520-shRNA-NC panels in Figure 7A of [3].
- The shVector panel in Figure 7A of [1] is similar to the NCI-H520 shRNA-NC panel in Figure 6B of [3] when rotated.
- In the Actin panels in Figure 1C, small bands/marks under the bands are present in the underlying image but are not present in the published images.
- In Figure 6B, the shALDOA-1 panel partially overlaps with the shALDOA-2 panel when rotated according to the corresponding author.
- The following inaccuracies/omissions were identified in the Methods section:
  ○ The xenograft tumor study is described as having 3 male mice per group, but the corresponding authors clarified that 2 male and 2 female mice were used per group.
  ○ 8% and 10% SDS-PAGE gels were used for western blot experiments, not 12% as stated.
  ○ Sequences of shRNAs against ALDOA and the non-targeting control are absent from the article. The corresponding authors provided the relevant sequences in S1 File.

The corresponding authors stated that data underlying all the results reported in the article are available, except for the raw image data underlying the western blots in Figure 5.

The corresponding authors confirmed that the same 2D gel image and cohort of 7 patients are reported in four articles [1–4], and each article focuses on a different aspect of the 2D gel results. They also reported that the control data in Figures 6A and 7A of this article [1] are identical to the data in [3], as the experiments were performed simultaneously using the same control samples. The figure legends for Figures 1, 6 and 7 are updated to acknowledge this data reuse, and the 2D gel image in Figure 1A has been replaced with a version that has software-analyzed annotation. The Editors remain concerned that a related study [2] that was under consideration at the time this article [1] was submitted was not declared, and that the reuse of data from this article [1] was not adequately acknowledged in subsequent publications.

The underlying image data for the actin panels in Figure 1C provided during discussions appear to contain bands/marks that are not present in the published image (S2 File). An updated version of Figure 1C is provided with this notice presenting the unadjusted images. The Editors remain concerned about the undisclosed beautification of images for publication, but consider that the original published panels accurately presented the results.

For Figure 6B, the corresponding authors reported that the shALDOA-2 panel is incorrect. They provided an updated Figure 6 in which the shALDOA-2 panel is replaced, and they provided underlying image data for the three samples presented in Figure 6B (S3 File).

The *PLOS ONE* Editors issue this Expression of Concern to notify readers of the above issues and relay the supporting data and updated figures provided by the corresponding authors.

**Figure 1:**
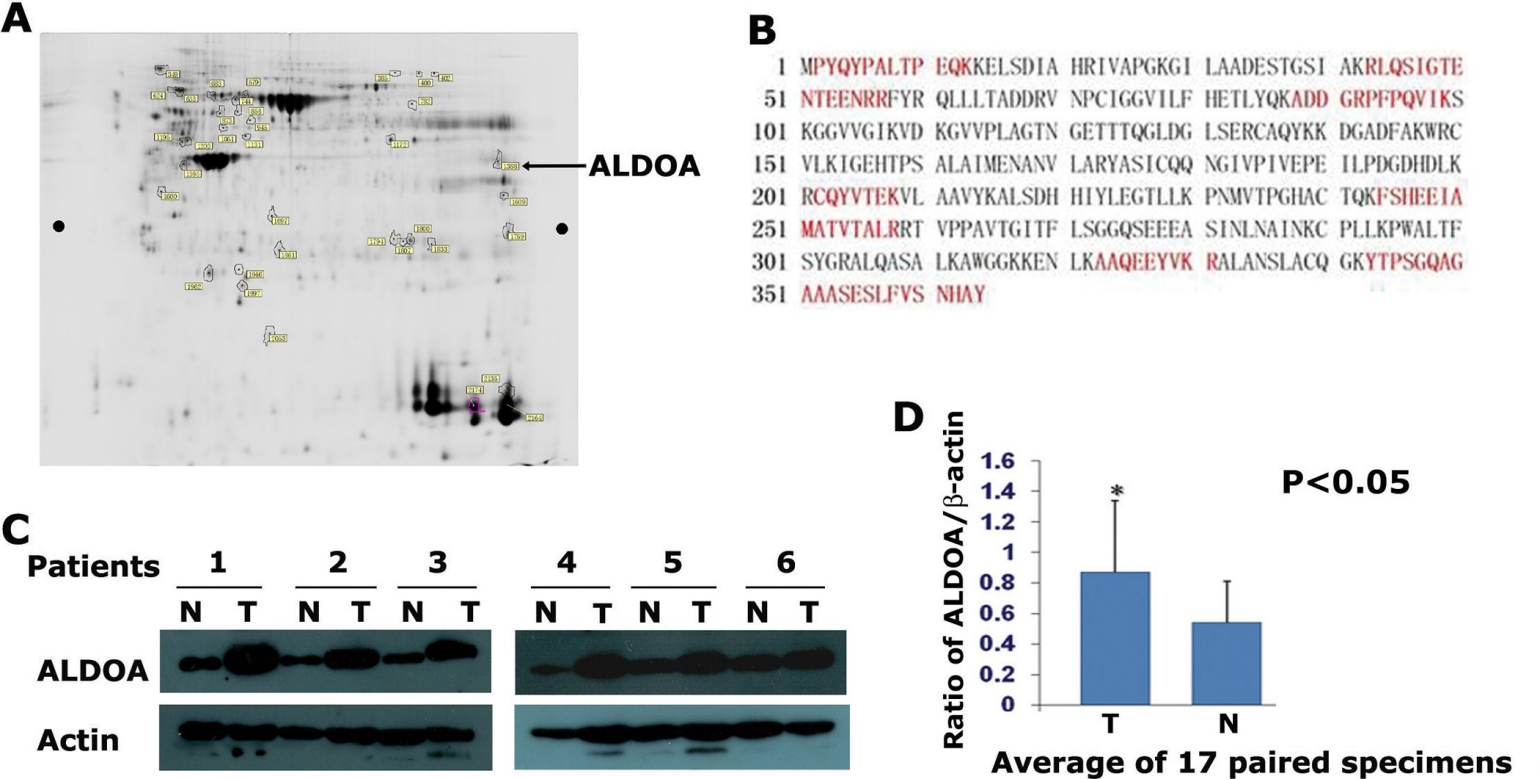
ALDOA is highly expressed in lung squamous carcinoma. (A) Proteomic analysis of the differentially expressed proteins from normal tissue and LSCC metastasis. Strictly selected 7 pairs of the matched LSCC specimens were subjected for 2D-DIGE and MS proteomic analysis. ALDOA was up-regulated 3.12-fold in metastatic LSCC tissues and 1.77-fold in non-metastatic LSCC tissues compared to adjacent normal tissues (the pointing arrow shows the ALDOA spots). The 2D gel electrophoresis image in Figure 1A has been replaced with a version that has software-analyzed annotation. (B) The amino acid residues highlighted in red were those detected by MS/MS analysis and counted for 36% sequence coverage of ALDOA. (C) Western blotting analysis of the ALDOA protein expression in 17 pairs of LSCC and matched adjacent normal tissues. Higher expression of ALDOA was observed in most of LSCC tissues examined. Data from 6 pairs of specimens were shown here. (D) Average of the ALDOA protein from 17 pairs of matched specimens. The relative expression values of ALDOA were 0.87±0.47 in carcinoma tissues and 0.54±0.27 in normal tissues. The level of Actin was used as control for normalization.

**Figure 6:**
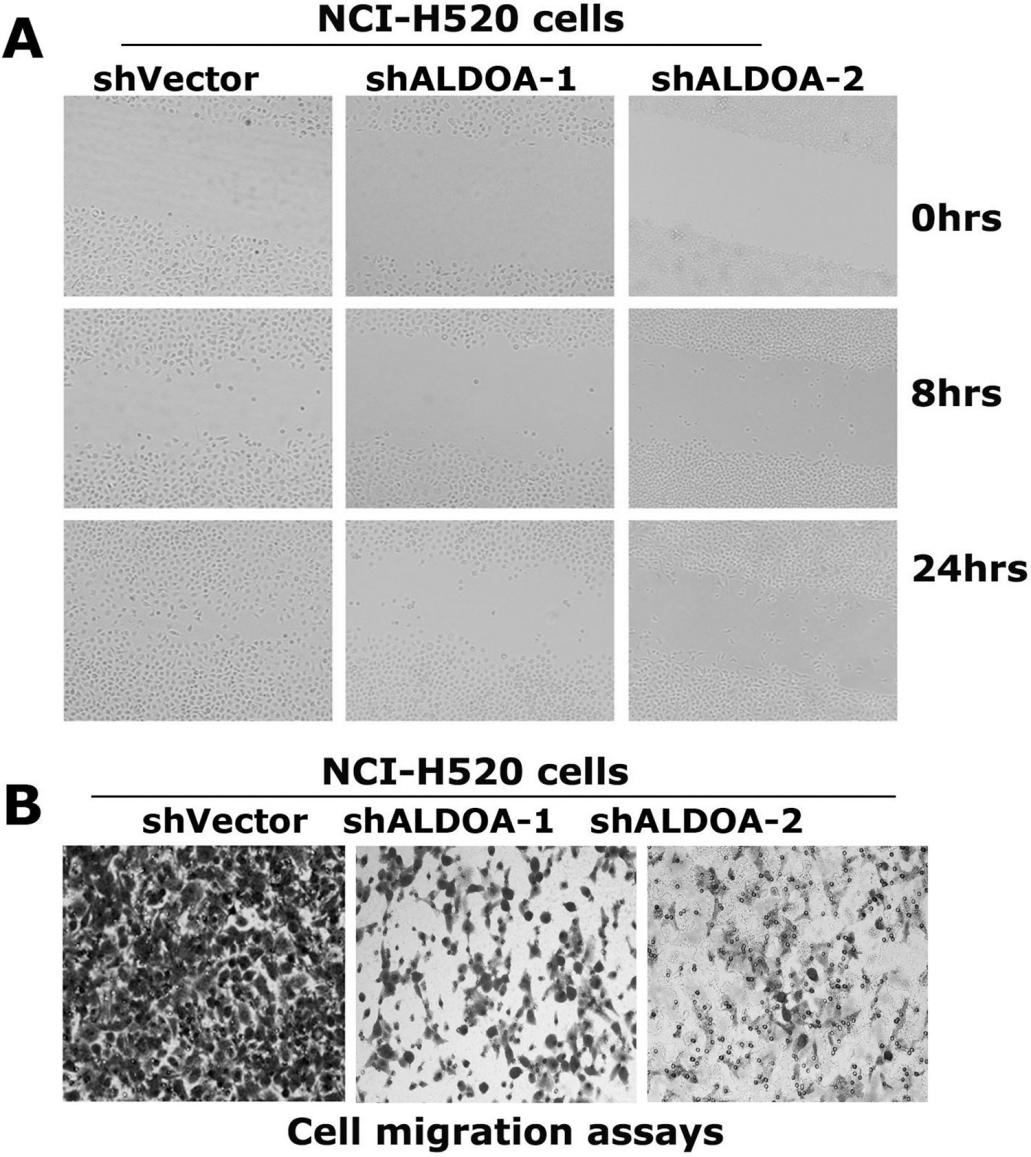
ALDOA is required for migration of the lung cancer cell NCI-H520. (A) Wound-healing assays of the resulting NCI-H520 cells in cultured plates. Cell were grown in petri dishes to 90% confluency and cut with a sterile 200-μl pipette tip. The resulting cells were continually cultured in serum-free medium and were photographed at 0, 8 and 24 hs post-scratching. The shVector panels in Figure 6A of [1] are reused as the NCI-H520-shRNA-NC panels in Figure 7A of [3]. (B) Cell migration assays using the transwell assays kits. 5×10^**5**^ cells per well were plated in the upper chambers of transwell plates with an 8-μm pore size membrane and media containing 10% FBS was placed in the lower chamber. After 24 hours of culture the cells on the upper surface of the filter were removed. The migrated cells were fixed in 70% methanol and stained with 0.1% crystal violet. The assays were performed in triplicates and at least 6 unbiased fields were counted per filter.

**Figure 7:**
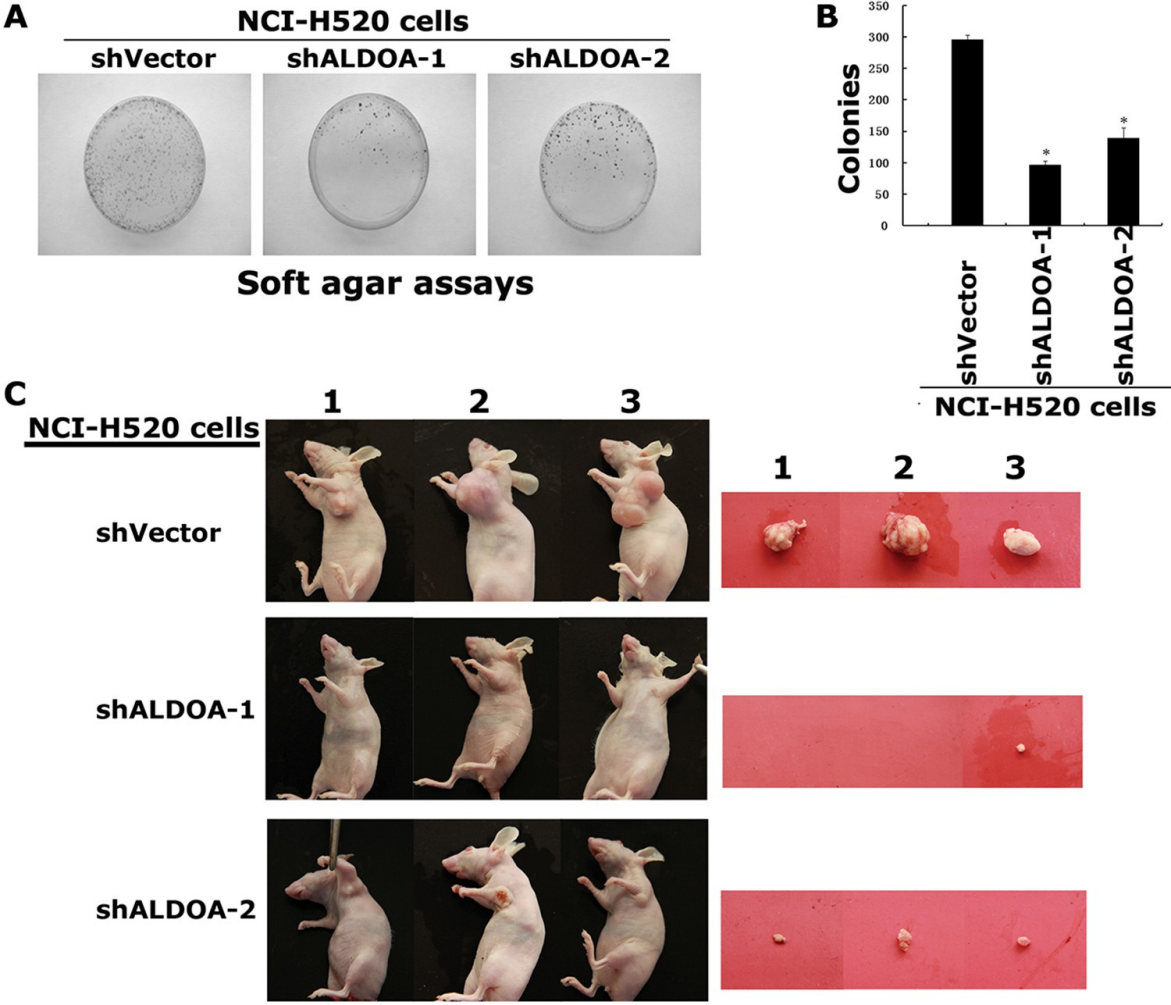
Depletion of ALDOA reduces the tumorigenicity of NCI-H520 cells. (A) Soft agar assays. The NCI-H520-siALDOA cells and NCI-H520-vector cells were suspended in single cell and cultured in soft agar to evaluate anchorage-independent colony formation. The experiments were done in triplates and repeated twice. The shVector panel in Figure 7A of [1] is reused as the NCI-H520 shRNA-NC panel in Figure 6B of [3]. (B) Data shows the average of the colonies formed by these NCI-H520 cells in triplicates. (C) The NCI-H520-shALDOA cells did not grow or only grow into very small tumors in the nude mice.

## Supporting information

S1 File Sequence information for shRNAs against ALDOA (shALDOA-a and shALDOA-2) and non-targeting control (shVector). (DOCX)

S2 File Image data underlying Figure 1C. (ZIP)

S3 File Image data underlying Figure 6B. (ZIP)
