# Supplementary material for: Expression of Concern: Fructose-Bisphosphate Aldolase A Is a Potential Metastasis-Associated Marker of Lung Squamous Cell Carcinoma and Promotes Lung Cell Tumorigenesis and Migration
Source: PLoS One. 2023 Apr 24;18(4):e0285076. doi: 10.1371/journal.pone.0285076 (PMC10124826; doi:10.1371/journal.pone.0285076)
Supplement: S1 File — (DOCX) [file pone.0285076.s001.docx]

S1 File. Sequence information for shRNAs against ALDOA (shALDOA-a and shALDOA-2) and non-targeting control (shVector).

shRNA-ALDOA-1:

5-GCCCTACCAATATCCAGCACT-3,

3-AGTGCTGGATATTGGTAGGGC-5

shRNA-ALDOA-2:

5-GGCGTTGTGTGCTGAAGATTG-3,

3-CAATCTTCAGCACACAACGCC-5

shVector:

5-GTTCTCCGAACGTGTCACGT-3,

3-ACGTGACACGTTCGGAGAA-5
