# Supplementary figures and images for: Expression of Concern: Fructose-Bisphosphate Aldolase A Is a Potential Metastasis-Associated Marker of Lung Squamous Cell Carcinoma and Promotes Lung Cell Tumorigenesis and Migration
Source: PLoS One. 2023 Apr 24;18(4):e0285076. doi: 10.1371/journal.pone.0285076 (PMC10124826; doi:10.1371/journal.pone.0285076)

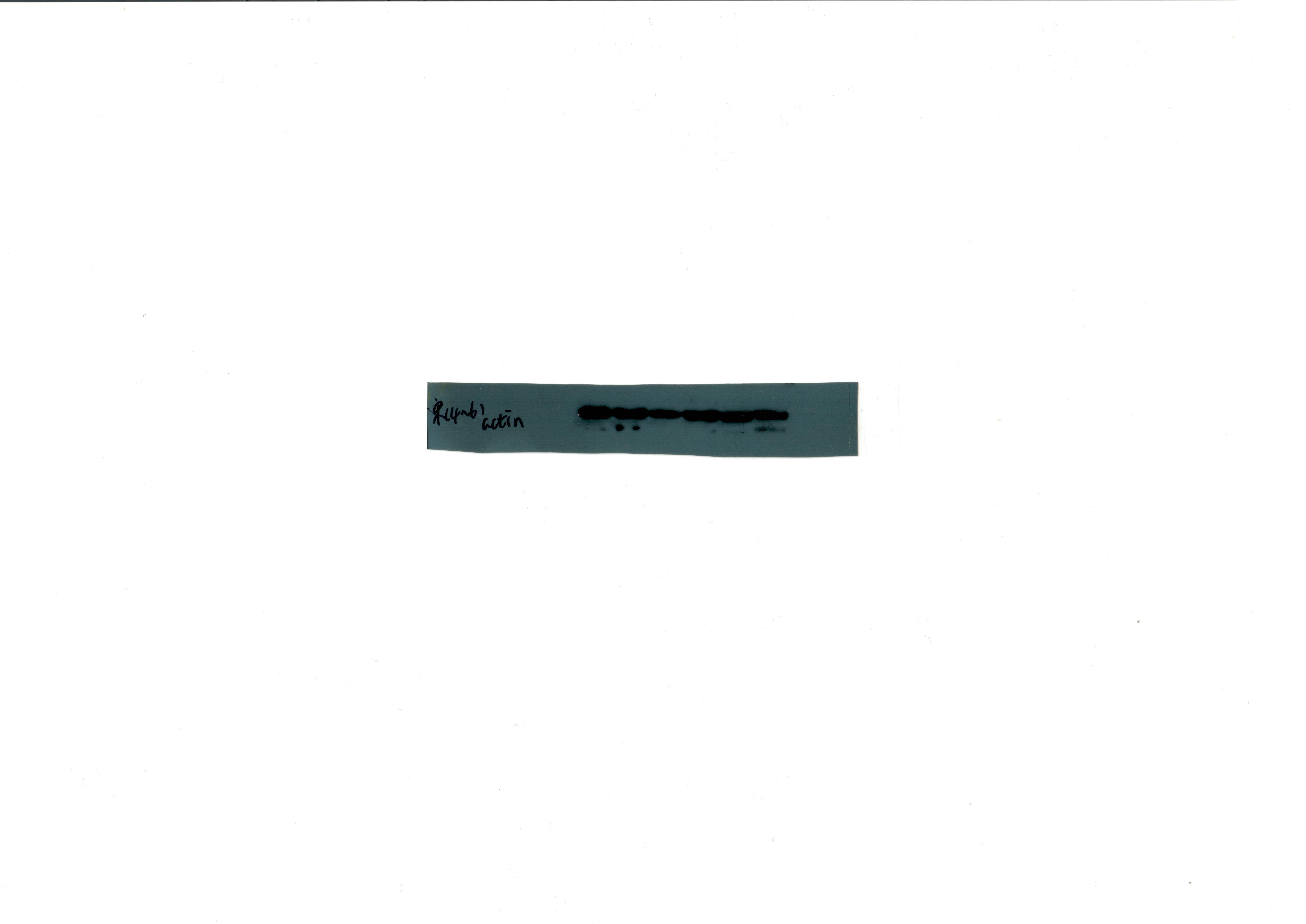

Supplement: S2 File — (ZIP) [file pone.0285076.s002.zip › S2 File. Raw image data underlying Figure 1C/Fig1C_1-3 Patients(N T) Actin WB.jpg]

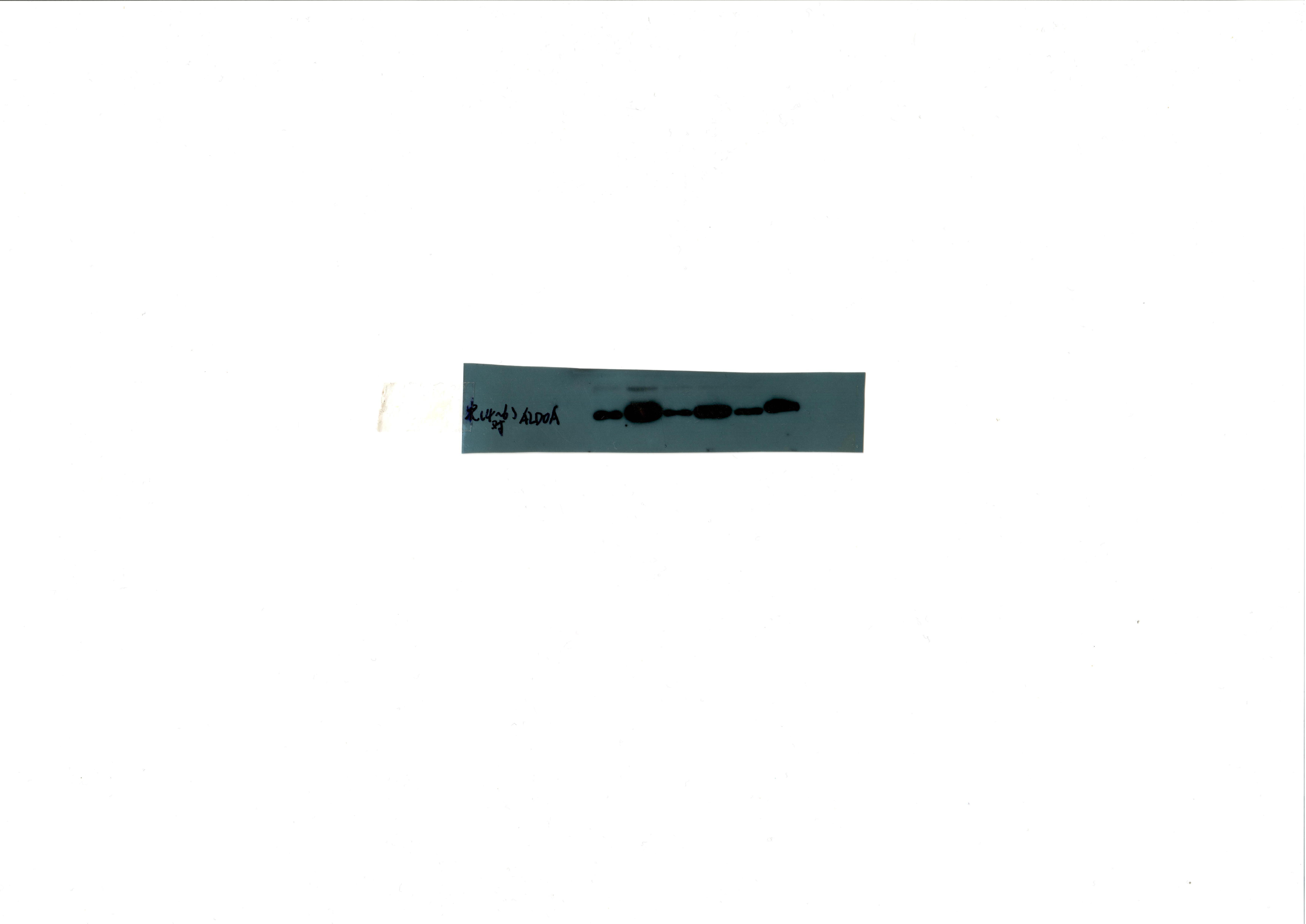

Supplement: S2 File — (ZIP) [file pone.0285076.s002.zip › S2 File. Raw image data underlying Figure 1C/Fig1C_1-3 Patients(N T) ALDOA WB.jpg]

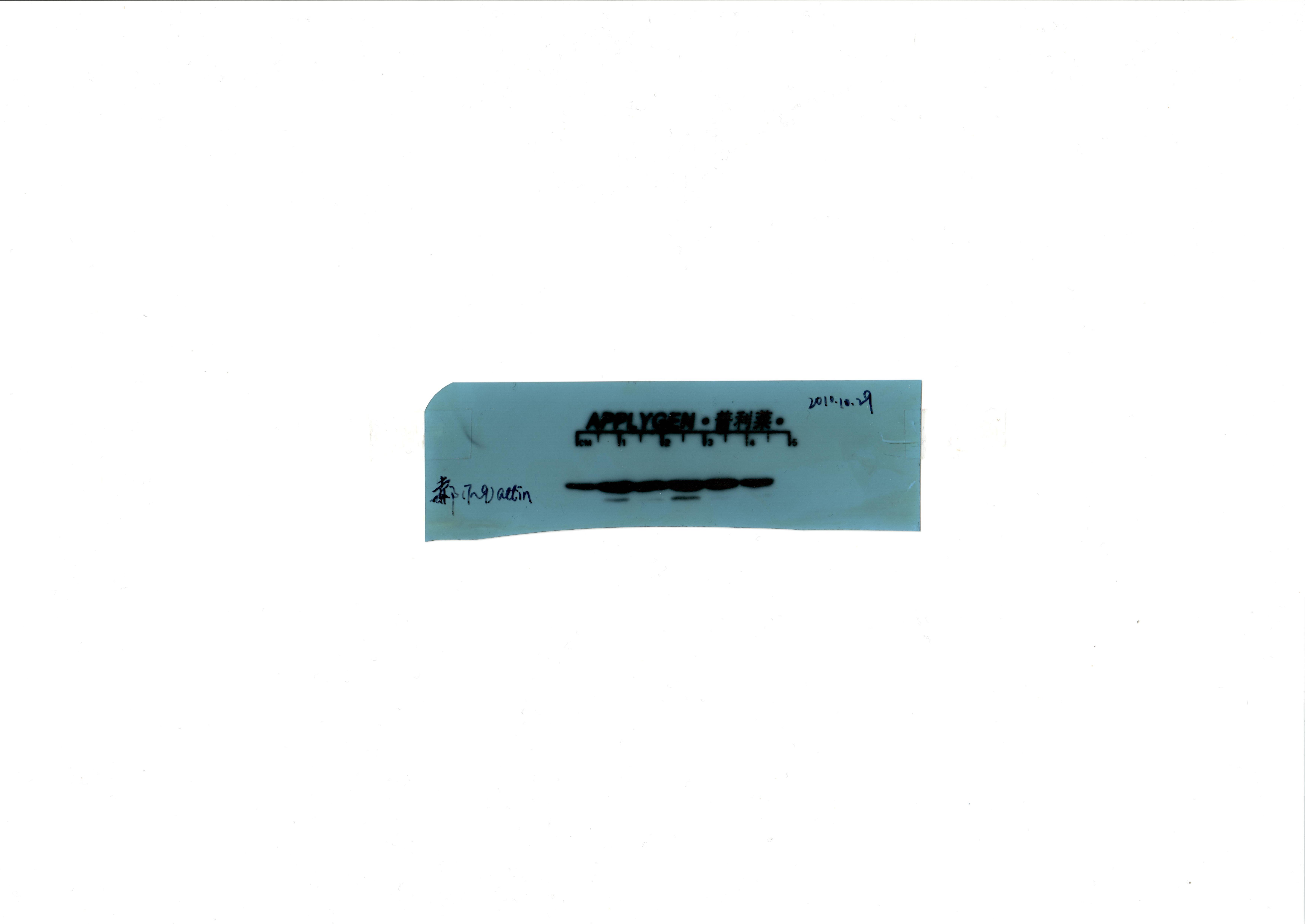

Supplement: S2 File — (ZIP) [file pone.0285076.s002.zip › S2 File. Raw image data underlying Figure 1C/Fig1C_4-6 Patients(N T) Actin WB.jpg]

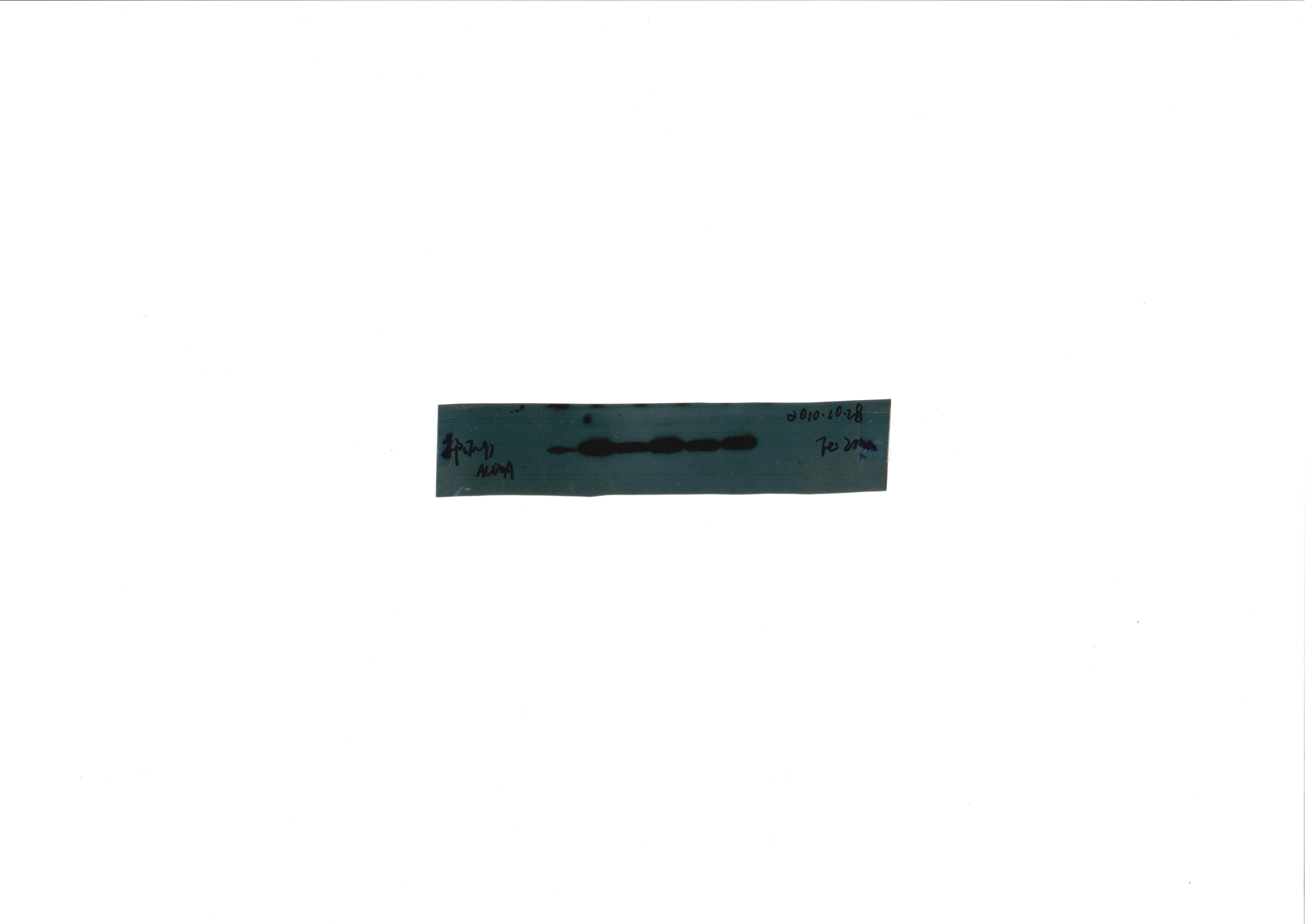

Supplement: S2 File — (ZIP) [file pone.0285076.s002.zip › S2 File. Raw image data underlying Figure 1C/Fig1C_4-6 Patients(N T) ALDOA WB.jpg]

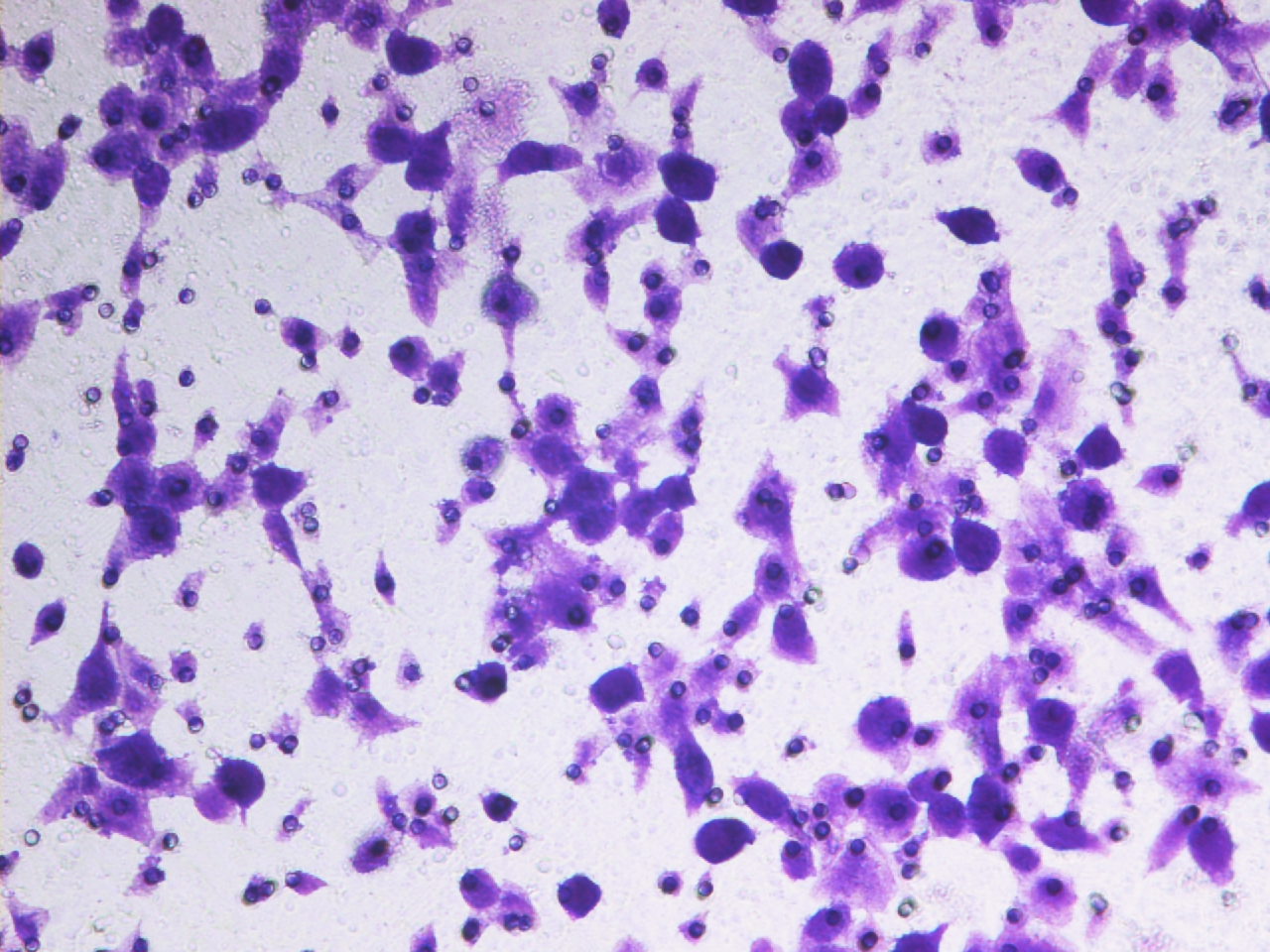

Supplement: S3 File — (ZIP) [file pone.0285076.s003.zip › S3 File/Fig 6B_NCI-H520-shALDOA -1.tif]

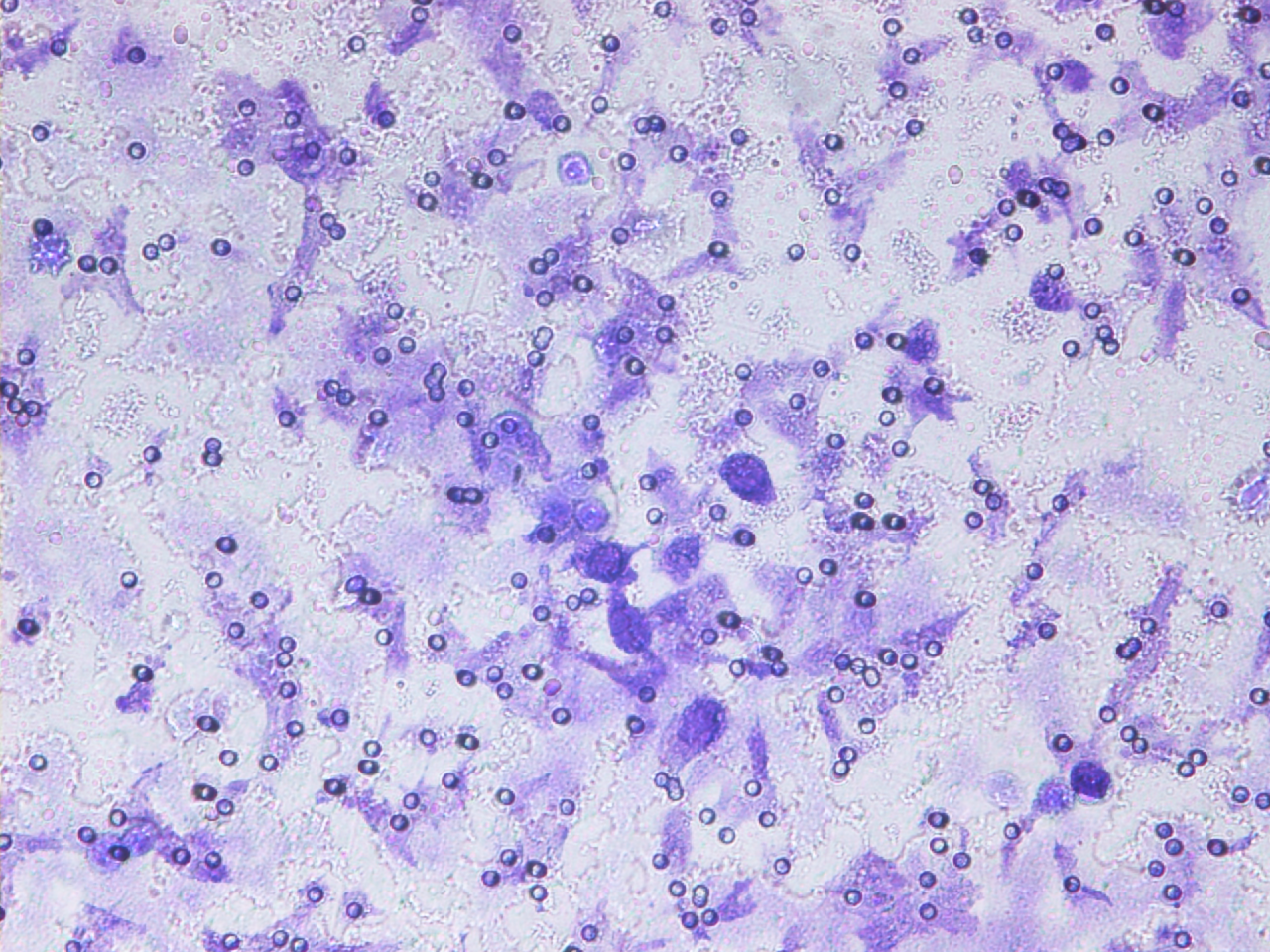

Supplement: S3 File — (ZIP) [file pone.0285076.s003.zip › S3 File/Fig 6B_NCI-H520-shALDOA -2.tif]

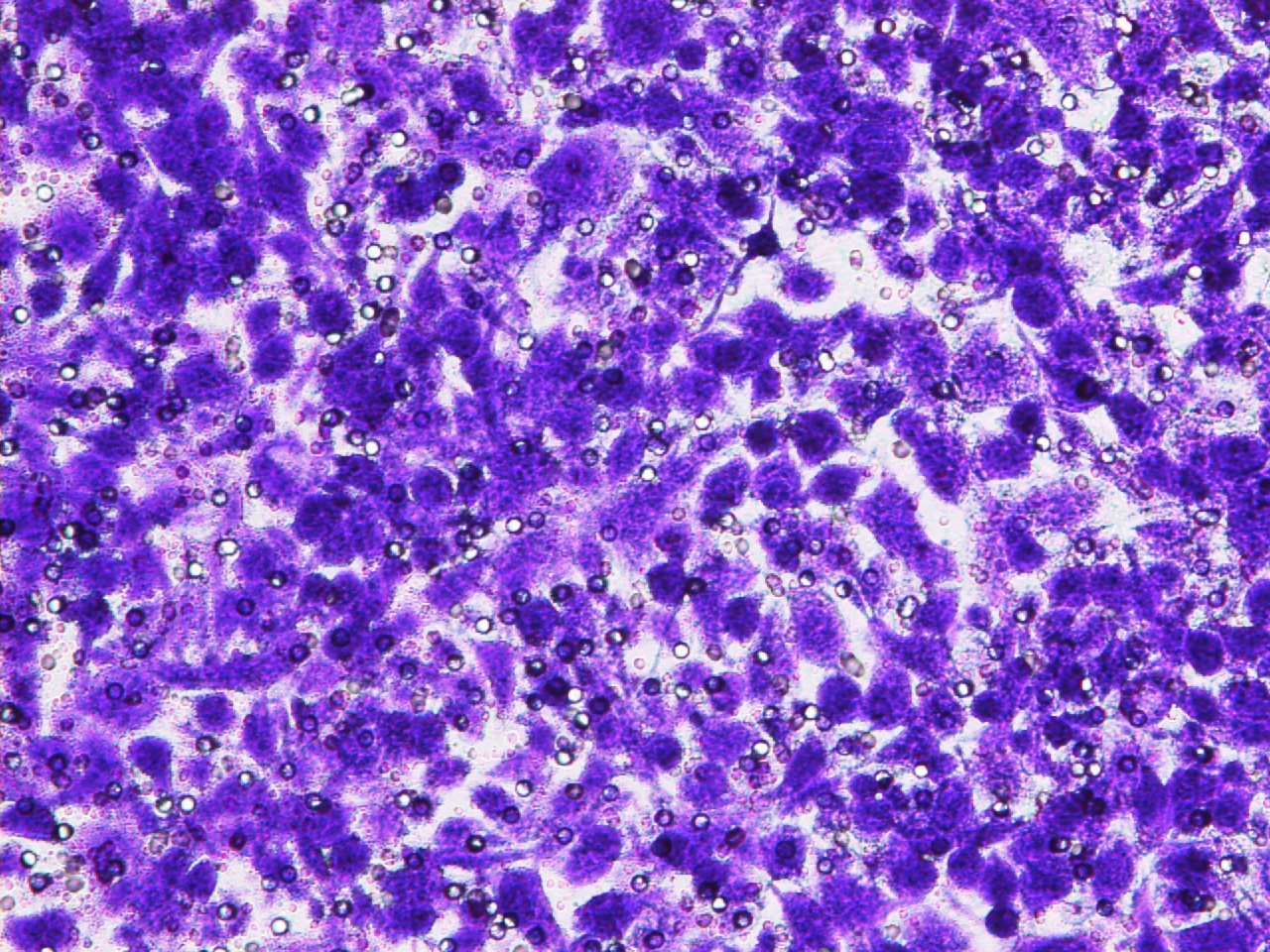

Supplement: S3 File — (ZIP) [file pone.0285076.s003.zip › S3 File/Fig 6B_NCI-H520-shVector.tif]
